# Effects of Hypopressive Techniques on the CORE Complex: A Systematic Review

**DOI:** 10.3390/healthcare13121443

**Published:** 2025-06-16

**Authors:** Pablo Hernandez-Lucas, Isabel Escobio-Prieto, Paloma Moro López-Menchero

**Affiliations:** 1 Department of Functional Biology and Health Sciences, Faculty of Physiotherapy, Universidade de Vigo, Campus A Xunqueira, s/n, 36005 Pontevedra, Spain; phernandez@uvigo.es; 2 Research Group HI10, 36005 Pontevedra, Spain; 3 Instituto de Biomedicina de Sevilla-IBiS (Hospitales Universitarios Virgen del Rocío y Macarena/CSIC/Universidad de Sevilla), 41092 Sevilla, Spain; 4 Departamento de Fisioterapia, Universidad de Sevilla, 41092 Sevilla, Spain; 5 Research Group CTS-1137, Neurological Physiotherapy Innovative Neurorehabilitation and Neurodevelopment Disorders, NEUROPHYSIUS, 41008 Seville, Spain; 6 Department of Physiotherapy, Occupational Therapy, Rehabilitation and Physical Medicine, University Rey Juan Carlos, 28922 Madrid, Spain; paloma.moro@urjc.es

**Keywords:** pelvic floor, pelvic floor disorders, abdominal muscles, abdominal CORE, low back pain, back pain, diaphragm and hypopressive exercise

## Abstract

The CORE complex refers to the muscles of the core region of the body, including the abdominal muscles, lower back muscles, and diaphragm. Among the various techniques aimed at improving CORE strength and functionality, abdominal hypopressive techniques have gained popularity. **Objectives**: To evaluate the available scientific literature on the effects of AHT on the CORE complex. **Methods**: A systematic search was conducted in January 2025 in PubMed, Web of Science, PEDro, Cochrane, ClinicalTrials.gov and Scopus. Only randomized controlled trials (RCTs) involving adults were included. Two reviewers independently selected studies and extracted data. The review was registered in PROSPERO (CRD-42023424933) and followed PRISMA guidelines. **Results**: Of 258 studies identified, 13 of them met the eligibility criteria for the final review. Showing the application of abdominal hypopressive techniques could have positive effects on the pelvic floor, transverse abdominis muscle, lumbar region, and diaphragm. The average methodological quality of the articles is 6.6 according to the PEDro scale. The risk of bias was high in 12 articles. **Conclusions**: Although the results show improvements in the CORE muscles after applying abdominal hypopressive techniques, further research is necessary to confirm these findings, given the insufficient methodological quality of the scientific literature and its high risk of bias.

## 1. Introduction

The lumbo-pelvic coxal complex, known as “CORE”, is a three-dimensional region of the body delimited by the diaphragm above, the abdominal muscles on the sides, the paravertebral and glutes behind, and the pelvic floor (PF) muscles below [1]. These structures work synergistically to stabilize the spine and regulate intra-abdominal pressure [1,2]. According to Panjabi’s model [3,4], core stabilization involves three interconnected subsystems: the passive system (comprising vertebrae, intervertebral discs, ligaments, and the passive components of muscles), the active system (consisting of core muscles providing dynamic support), and the neural system (responsible for processing and coordinating motor responses). These subsystems interact continuously to ensure postural control and efficient movement.

Beyond spinal stabilization, the CORE complex also plays a vital role in postural control, force transmission between the upper and lower limbs, motor control, and injury prevention. As such, optimizing the CORE function is crucial for both athletic performance and rehabilitation.

Among the techniques used to target deep musculature and reduce intra-abdominal pressure, the abdominal hypopressive technique (AHT) has gained in popularity. The AHT is a series of postural and respiratory exercises originally developed to improve pelvic floor function and abdominal tonicity. These exercises involve specific body positions combined with expiratory apnea and diaphragmatic aspiration, aiming to generate a reflex activation of deep stabilizing muscles, such as the PF and transversus abdominis (TrA) [5,6,7,8,9,10]. However, despite its growing use in clinical practice, scientific evidence on the physiological effects and functional impact of the AHT remains limited and methodologically inconsistent, particularly regarding its influence on the CORE complex as a whole.

Previous systematic reviews have mainly focused on women with pelvic floor dysfunction [11,12], without comprehensively addressing the effects of the AHT on all CORE-related regions or including male participants. Given that muscle activation patterns during AHT exercises have also been demonstrated in men [10], and that the AHT has shown potential benefits beyond the pelvic floor, such as in the lumbar spine and diaphragm [13], a broader evaluation is warranted.

Therefore, this systematic review aims to analyze the effects of abdominal hypopressive techniques on the CORE complex, considering evidence from randomized controlled trials in both men and women, and examining outcomes across the pelvic floor, abdominal wall, lumbar region, and diaphragm. This broader scope distinguishes the present review from prior literature and seeks to provide a more comprehensive understanding of the AHT’s potential clinical applications.

## 2. Materials and Methods

### 2.1. Design

This research was prospectively registered in PROSPERO (code: CRD-42023424933), following the guidelines established by PRISMA (Preferred Reporting Items for Systematic Reviews and Meta-analyses) [14], as well as the reporting standards in PRISMA in Exercise, Rehabilitation, Medicine and Sports Sciences (PERSIST) [15] and the recommendations of the Cochrane Collaboration [16].

In January 2025, a comprehensive search for publications was conducted in the following databases: Web of Science, PubMed, PEDro, Cochrane, ClinicalTrials.gov, and Scopus. A variety of combinations were used with the following Medical Subject Headings (MeSH): “pelvic floor”, “pelvic floor disorders”, “abdominal muscles”, “abdominal CORE”, “low back pain”, “back pain”, and “diaphragm.” Additionally, the following free-text terms were included: “hypopressive”, “hypopressive exercise”, and “hypopressive abdominals.”. Appendix A: Table A1 presents the search equations used in each database, which were based on a specific PICO question. P (Population) comprises adults (≥18 years) with symptoms associated with CORE complex dysfunctions, such as pelvic floor disorders, urinary incontinence, low back pain, postural alterations, or post-surgical conditions. I (Intervention) involves the application of the AHT, either as a standalone treatment or within a multimodal approach. C (Comparison) is defined as no intervention, placebo, or other active treatments, such as pelvic floor muscle exercises, massage therapy, biofeedback, or electrostimulation. O (Outcomes) include pelvic floor strength, symptom reduction (e.g., incontinence, pain), postural control, respiratory function, and quality of life. S (Study Design) selected randomised controlled trials and controlled clinical trials published in peer-reviewed journals. Studies were included if they applied the AHT as a central or complementary intervention, assessed at least one predefined outcome, and were available in full text in English or Spanish. Exclusion criteria comprised case reports, reviews, editorials, conference abstracts, studies involving minors or animals, or lacking detailed information on AHT implementation. The search included studies in English and Spanish, with no restrictions on publication status; no filters were applied during the search, and grey literature was not included.

### 2.2. Study Selection

After eliminating duplicates, two authors (P.M.L.-M.; P.H.-L.) continued reading the titles and abstracts, eliminating the studies that did not agree with the objective of the systematic review. The Kappa coefficient between the first and second reviewers was 0.9, indicating almost perfect agreement. After completing this process, each of them was analyzed applying the following inclusion criteria for the selection studies: (i) men and women, (ii) between 18 and 60 years of age, (iii) randomized controlled studies, and (iv) intervention with the AHT. The following exclusion criteria were also applied: (i) variables related to the CORE were not analyzed, and (ii) the study was not published. After screening the results based on the inclusion criteria using a personalized Microsoft Excel table, the articles for this review were obtained.

### 2.3. Data Extraction

Two reviewers (P.M.L.-M.; P.H.-L.) extracted the following data for analysis: demographic information (title, authors, journal and publication year), sample characteristics (age, sex and number of participants), specific parameters of the study (duration of the intervention, adverse events and exercise methods), and the results obtained (variables analyzed, instruments used and follow-up time). Tables were used to describe both study characteristics and extracted data.

### 2.4. Quality Assessment

The PEDro scale [17] was used to evaluate the quality of these studies and the RoB scale (Risk of Bias) [18] tool was applied to evaluate the risk of bias. Two reviewers (P.M.L.-M.; P.H.-L.) applied the PEDro and RoB scales. In cases where the reviewers disagreed, a third reviewer (I.E.-P.) was consulted to make the final decision regarding the inclusion or exclusion of a study from the analysis.

## 3. Results

**Figure 1:**
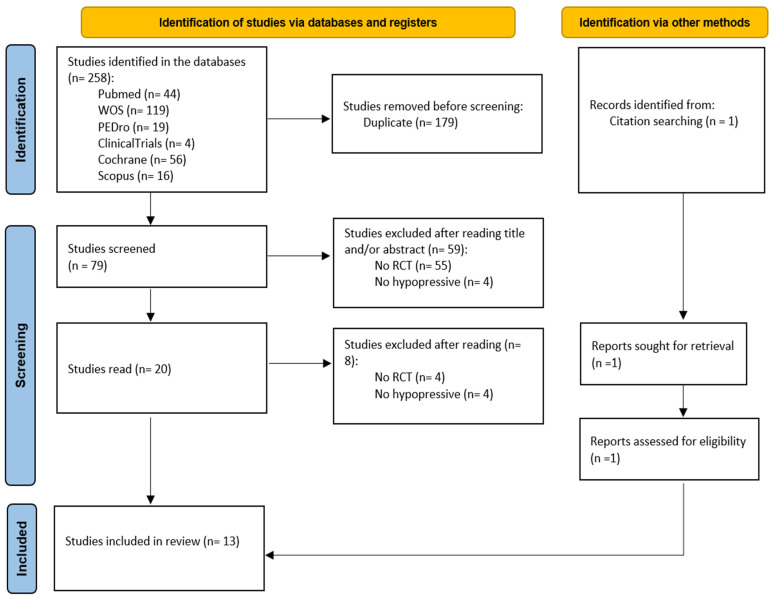
Flow chart. Note: This is a flow chart according to PRISMA [14].

Out of 258 search results, 79 studies were considered eligible for inclusion after removing duplicates and reviews. Among the 79 articles screened, 59 were excluded after abstract and title screening. Of the 20 full-text articles assessed for eligibility, eight were excluded. Thirteen articles were ultimately included in the synthesis [19,20,21,22,23,24,25,26,27,28,29,30] and a new article was added in the author search [31] (Figure 1).

### 3.1. Methodological Quality of the Studies and Risk of Bias

The average score of all articles included in this review was 6.6 points, which indicated a moderate methodological quality (Table 1) [17]. The articles with the lowest score obtained five points [23,29] and those with the highest score attained eight points [22,27].

**Figure 2:**
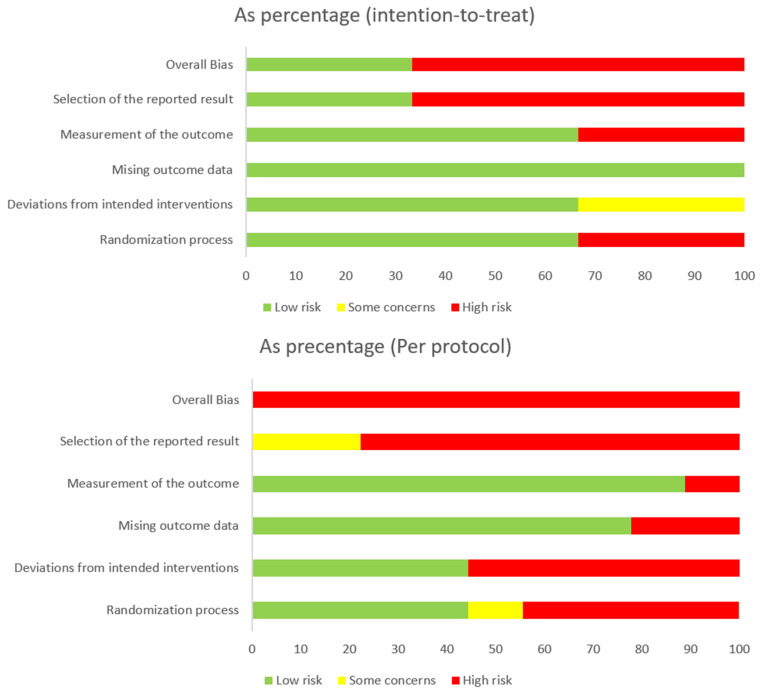
Risk of Bias Graph (summary bar chart by domain).

**Figure 3:**
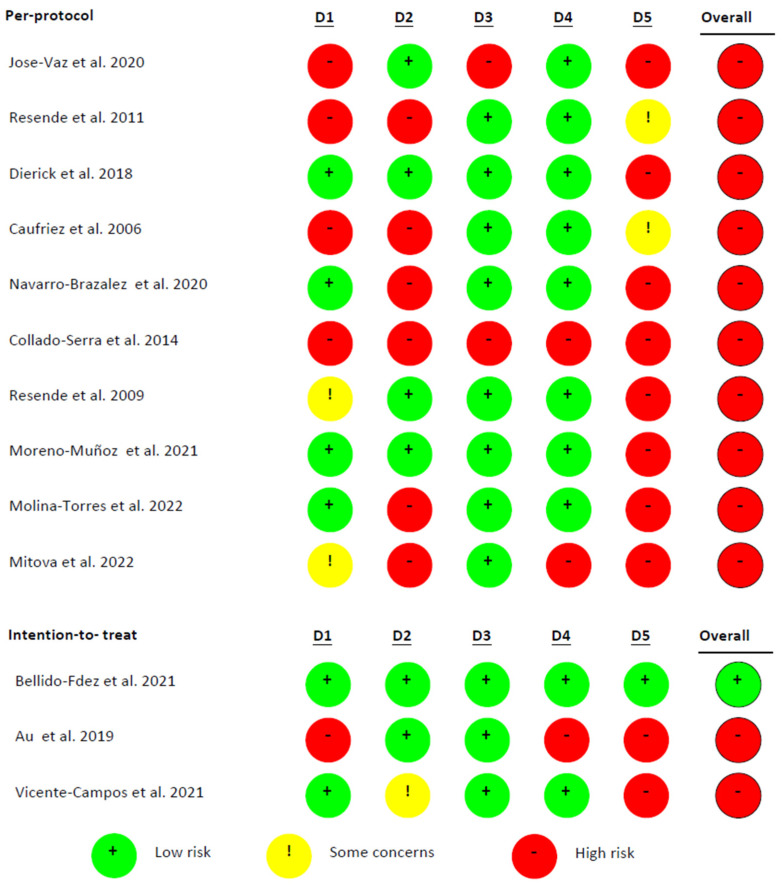
Risk of Bias Summary (per-study and per-domain assessment). Note: D1: randomization process; D2: deviations from the intended interventions; D3: missing outcome data; D4: measurement of the outcome; D5: selection of the reported result [19,20,21,22,23,24,25,26,27,28,29,30,31].

According to the Cochrane Collaboration Tool to assess the RoB, of the 13 studies, three are intention-to-treat [22,25,28] and nine are per protocol [19,20,21,23,24,27,29,30,31]. One of them presents a low RoB [22] and the rest show a high RoB [19,20,21,23,24,25,26,27,28,29,30,31] (Figure 2 and Figure 3). 

### 3.2. Participants

A total of 992 people participated in the 13 studies [19,20,21,22,23,24,25,26,27,28,29,30,31], and the mean age was 42.4 years, with a range from 23 to 63 years. It is worth mentioning that three articles do not describe the age or the participants [23,29,31] (Table 2).

### 3.3. Interventions

In the 13 articles included in this review, the AHT intervention group could be used as a single technique [19,22,24,26,27,28,29,30,31] or combined with PF exercises [20,21,23,24,25,29] or with massage therapy [22]. It is compared with a control group in which they have no intervention [20,23,24,26,27,28], a PF exercise group [19,20,24,25,29,30], an electro-biofeedback group [21], a massage therapy group [22], and a postural training group [31] (Table 3).

The AHT exercises were carried out according to these guidelines: self-elongation, neutral position of the pelvis, ankle dorsiflexion, knee flexion, activation of the shoulder girdle muscle, three respiratory cycles with lateral costal breathing and slow deep exhalation (inspiration, maximum exhalation), and maintaining breathing after expansion of the rib cage (diaphragmatic aspiration), with a learning respiratory pattern of complete expiration and diaphragmatic aspiration [20,21,24,30]. In two articles, prior learning is not indicated [22,23]. In one of the articles, diaphragmatic aspiration was not performed, but abdominal contraction was performed during expiratory apnea [29] (Table 3).

The AHT exercises were static with different positions of the limbs in the supine, sitting and biding positions [19,20,24], and with quadrupedy [21]. In others, a dynamic progression was made from the standing to supine positions [26,27,30,31]. In the remaining studies, the type of technique is not specified [22,23,25,28,29]. The duration of the treatment varied between 3 weeks [21] and 25 weeks [25], with an average of 14 weeks. On average, three sessions were conducted per week, with each session lasting approximately 38 min. Treatment intensities ranged from brief interventions of 20 min twice per week to an intensive regimen of up to 45 min, five times per week over several months.

### 3.4. Effects

Regarding the articles that assess PF strength [19,20,21,24,27,30], it is observed that the AHT group does not increase the strength variables compared to the PF exercise group [19,21] or that there is no significant difference between both groups [20,24,30]. However, the AHT group significantly increases the strength variables compared to the control group [20,27] and compared to baseline levels [19,20,21,24,27,30].

In five articles, urinary incontinence (UI) is assessed [19,23,25,27,29] and a significant decrease in symptoms is observed in the AHT group compared to baseline values [19,23,25,27,29] and compared to the control group [23,27]. But regarding the PF muscle exercise (PFMX), one article shows improvement in UI compared to the AHT [19] and in another, there are no significant differences between groups [25]. Finally, Mitova et al., 2022, obtained significant changes in UI in favor of the AHT group in combination with Kegel exercise, compared to AHT and Kegel exercise groups used alone [29]. In the articles that assess the abdominal region [24,26,29], it is observed that the AHT group improves the variable of postural control [26,29] and abdominis transversus (TrA) strength compared to baseline levels [24,26,29] and the control group [24,26,29].

Two articles assess lumbar flexibility [22,31], and it is observed that the AHT group improves the mobility variable compared to baseline levels [22,31] and compared to the postural training group [31]. Furthermore, the AHT group in combination with massage therapy significantly improves disability compared to only using massage therapy or only performing AHT exercises [22].

Finally, only one article assesses the effect on the diaphragm [28], in which they observed that the AHT group improves the variables of diaphragmatic thickness and forced inspiration compared to basal levels and the control group.

## 4. Discussion

The main objective of this review was to analyze the available scientific literature on the effects of the AHT on CORE. The results of the articles included in this systematic review suggest that the AHT could have positive effects at the level of the PF, TrA, lumbar region and diaphragm.

In this systematic review, six studies assessed the strength of the pelvic floor (PF) [19,20,21,24,27,30]. Although the AHT group showed significant improvements compared to baseline levels in most studies [19,20,21,24,27,30], the true between-group differences were more limited. Specifically, the AHT group did not show superior improvements compared to the pelvic floor muscle exercise (PFMX) group in several studies [19,21], and no significant differences were found between these two interventions in others [20,24,30]. For example, Resende et al. (2012) reported no significant advantage in adding the AHT to PFMX compared to performing PFMX alone. Similarly, Jose-Vaz et al. (2020) found greater improvements in PF strength variables in the PFMX group compared to the AHT group. However, the AHT group did demonstrate significantly better outcomes when compared to control groups [20,24,27], which suggests a potential benefit of the AHT in the absence of active intervention. This may be due to the reflexive activation of the musculature during AHT exercises, promoted by postural guidelines such as axial self-elongation, shoulder girdle activation, and a neutral pelvis [32,33]. These elements, combined with expiratory apnea and rib cage expansion, generate negative pressure in the abdominal cavity that may involuntarily activate type I slow-twitch fibers of the PF and abdominal muscles, enhancing muscle tone and endurance [30]. In contrast, PFMX involves voluntary contractions that mainly recruit type II fast-twitch fibers, which are associated with greater force generation [30].

In this work, five articles evaluate UI [19,23,25,27,29]. They show significant improvements in the AHT group compared to baseline values [19,23,25,27,29] and compared to the control group [23,27]. Nevertheless, regarding the PFMX group, the results are ambiguous; one article shows improvement in stress urinary incontinence (SUI) compared to the AHT [19] and, in another article, there are no significant differences between groups [25]. Furthermore, Mitova et al., 2022, obtained significant improvements in UI in the AHT group in combination with Kegel compared to groups using only AHT or Kegel exercises [29]. Similarly, leading clinical guidelines suggest conservative treatment using therapeutic exercises and lifestyle modifications as the first-line intervention [34].

Only three articles that assess the strength of the TrA [24,26,29] obtained significant improvements in all of them [24,26,29]. This may be due to the fact that the activation of the shoulder girdle and the neutral pelvis position are related to the activation of the CORE [35]. Furthermore, axial self-elongation and abdominal activation are strongly related due to optimal spinal alignment, activation of deep CORE muscles, balanced distribution of intra-abdominal pressure, muscle synergy, improved body awareness and reflex activation [36]. Other authors pointed out a decrease in waist circumference measurement after the intervention with the AHT, associating this effect with an activation of TrA [37,38,39].

Two articles [22,31] assessed lumbar flexibility, obtaining a significant increase in the mobility of this posterior region of the CORE in both studies [22,31]. Furthermore, the AHT group in combination with massage therapy significantly improved disability compared to only using massage therapy or only performing AHT exercises [22]. Along the same lines, the mobility of the lumbar region was evaluated by Ria et al., 2014, showing improvement after an AHT session [37]. On the other hand, the mobility of the lumbar region and TrA strength are important to address low back pain [40]. Previous research also relates AHT to improvements in pain and disability associated with low back pain [13,41].

Only one of the articles in our review [28] assessed the diaphragm of subjects performing AHT exercises and both diaphragmatic thickness and forced inspiration [28]. The benefits of these parameters could be explained by the sustained eccentric contraction of the diaphragm during AHT performance. In a different investigation, another respiratory parameter significantly increased in participants who performed AHT exercises—peak expiratory flow [39]. This means that the abdominal muscles contracted more to expel air more quickly [42]. This is related to the three articles that assessed the increase in TrA strength after performing AHT exercises [24,26,29].

The results obtained in the articles included in this systematic review must be interpreted with caution due to the following reasons: the high heterogeneity of the protocols; the insufficient methodological quality according to the PEDro scale, and 12 of the 13 articles present a high risk of bias [19,20,21,23,24,25,26,27,28,29,30,31]. Furthermore, it should be noted that the duration of the AHT interventions varied substantially across the included studies (ranging from 3 to 26 weeks), which may influence the magnitude of the reported effects. In addition, there were methodological differences regarding the use of static versus dynamic techniques, as well as the application of complementary therapies (such as pelvic floor muscle training, massage therapy, or electrostimulation). Several studies also lacked key participant data (e.g., age or baseline characteristics), which weakens the interpretability and comparability of the results. This variability limits the generalizability of the findings and hinders the identification of the specific component responsible for the observed effects. Moreover, due to the clinical and methodological heterogeneity of the included studies, it was not possible to conduct a meta-analysis, which restricts the statistical strength and precision of the overall conclusions.

Therefore, further research is needed to analyze the effects of the AHT in the CORE region, with larger samples and long-term follow-ups that help confirm these initial findings. Given the generally high risk of bias and methodological limitations in the included studies, the current evidence should be considered preliminary. The findings regarding the effectiveness of the AHT remain mixed, with some trials showing improvements and others reporting no significant differences compared to established interventions, such as pelvic floor muscle training. To advance this area of research, future studies should adopt standardized AHT protocols with clearly defined parameters, include larger and more diverse populations, and incorporate extended follow-up periods. High-quality randomized controlled trials with blinded assessments and rigorous methodological designs are essential before any firm clinical recommendations can be made.

Given the overall high risk of bias and methodological limitations identified in most of the included trials, the current evidence should be interpreted as preliminary. While some studies report positive effects of the AHT on PF strength, TrA activation, lumbar mobility, and diaphragm function, the findings are mixed and often inconsistent between studies. Notably, several trials failed to demonstrate superiority of the AHT over conventional pelvic floor muscle training. Therefore, the heterogeneity of protocols and the conflicting outcomes across studies highlight the urgent need for well-designed, high-quality randomized controlled trials before clinical recommendations can be made with confidence.

## 5. Conclusions

The results of the articles included in this systematic review suggest that the AHT could have positive effects at the level of the PF, TrA, lumbar region and diaphragm. However, the methodological quality of the scientific literature is preliminary and presents high risk of bias. Therefore, further research is necessary to confirm these findings.

## Figures and Tables

**Table 1:** The methodological quality of the included studies.

Author	1 *	2	3	4	5	6	7	8	9	10	11	Score
Au et al. (2020) [25]	N	Y	Y	Y	N	N	N	N	Y	Y	Y	6
Bellido-Fdez et al. (2022) [22]	Y	Y	Y	Y	N	N	Y	Y	Y	Y	Y	8
Caufriez et al. (2006) [31]	Y	Y	Y	N	Y	N	Y	Y	N	Y	Y	7
Collado-Serra et al. (2013) [23]	Y	Y	N	Y	N	N	N	Y	N	Y	Y	5
Dierick et al. (2018) [21]	Y	Y	N	Y	N	Y	Y	Y	N	Y	Y	7
Jose-Vaz et al. (2020) [19]	Y	Y	Y	Y	N	N	Y	N	N	Y	Y	6
Mitova et al. (2022) [29]	N	Y	N	Y	N	N	N	Y	N	Y	Y	5
Molina-Torres et al. (2023) [27]	Y	Y	Y	Y	Y	N	Y	Y	N	Y	Y	8
Moreno-Muñoz et al. (2021) [26]	Y	Y	Y	Y	N	N	Y	Y	N	Y	Y	7
Navarro-Brazález et al. (2020) [30]	Y	Y	Y	Y	N	N	Y	Y	N	Y	Y	7
Resende et al. (2010) [24]	Y	Y	Y	Y	N	N	Y	Y	N	Y	Y	7
Resende et al. (2012) [20]	N	Y	N	Y	N	N	Y	Y	N	Y	Y	6
Vicente-Campos et al. (2021) [28]	Y	Y	N	Y	N	N	Y	Y	Y	Y	Y	7

(*) This item pertains to external validity and does not contribute to the overall score; (Y = Yes); (N = No). (1) Eligibility criteria specified; (2) Subjects randomly allocated to groups; (3) Concealed allocation; (4) Groups were similar at baseline; (5) Blinding of all subjects; (6) Blinding of all therapists; (7) Blinding of all assessors; (8) Measures obtained from more than 85% of subjects allocated to groups; (9) Subjects received treatment or control condition as allocated, or intention-to-treat analysis; (10) Between-group statistical comparisons reported for at least one outcome; (11) Both point measures and measures of variability were reported.

**Table 2:** Study characteristics.

Authors	Observed Area	Sample	Intervention	Supervisor	Age	Period	Measures	Results
Jose-Vaz et al. (2020) [19]	EUI	130	G1 (n = 36): AHT G2 (n = 37): PFMX	PST PF	G1: 56.9 ± 11.5 G2: 53.4 ± 11.6	12 weeks G1: 3 phases/4 weeks each; training 50′ G2: 3 series 8–12 contraction max/session 6” contraction	7-day voiding diary, ICIQ-SF, MogS, manometry.	Significant improvements in both groups, in PF strength variables compared to baseline values. G2 significant improvements in strength variables compared to G1. G1 significantly improves EUI compared to baseline values. G2 significantly improves EUI compared to baseline values. G2 improves EUI results significantly compared to G1.
Resende et al. (2012) [20]	PF	58	G1 (n = 21): PFMX G2 (n = 21): APMFX G3 (n = 16): control	PST urogynecological	G1: 56.7 ± 10.7 G2: 51.9 ± 7.4 G3: 58.7 ± 0.4	12 weeks Daily	PF Strength: MOGS endurance. SEMG muscle activity.	G1 and G2 improved significantly in the PF strength variables compared to baseline values and compared to G3. There were no significant differences in muscle strength or activity between G1 and G2. The resistance in G1 was significantly higher than G2. G1 was significantly greater tan G3 in strength resistance and SEMG. G2 was significantly superior to G3 at the 3-month evaluation in all strength variables.
Dierick et al. (2018) [21]	Puborectal and iliococcygeal	24	G1 (n = 9): APMFX G2: (n = 8): BFB PFEE	PST PF	G1: 22.9 ± 1.2 G2: 23 ± 2	10–11months 270′ (9sessions/30′) 3 sessions/week for 3 weeks	MOGS: PF strength. rPR.iPR. PB idle tone. DA PDFI-20 and PDIQ-7. MRI: signal intensity straight-media vagina-media vagina at 3 and 9 volumes	Significant improvements in iPR and PB strength in both groups compared to baseline values. There were no differences in strength. PF resting tone nor in diaphragmatic aspiration in both groups compared to baseline values. G2 significantly improves dPR compared to baseline values. G1 did not obtain differences in rPR compared to baseline values. G2 significant improvement in dPR strength compared to G1. On MRI, the volumen of the IL and the signal intensity of the PR decreased significantly compared to baseline values in both groups. In the rest of the variables there were no significant differences.
Bellido-Fdez et al. (2021) [22]	NSLBP	60	G1 (n = 20): massotherapyG2 (n = 20): AHT G3 (n = 20): massotherapy + AHT	PST	G1: IQR 43 G2: IQR 27 G3: IQR 34	5 weeks 8 interventions of 30′	ODI. NRS. SF-12 and Schober’s test	There were significant improvements in all groups 4 weeks pre/post intervention compared to baseline values in disability; pain; lumbar mobility and the physical component of quality of life. G2: significant improvements in the mental component of quality of life compared to baseline values at the end of the intervention. G3 significantly improved disability compared to G1 and G2. In the rest of variables there were no significant differences.
Caufriez et al. (2006) [31]	Back	29	G1: AHT G2: posture training	-	-	10 weeks 1h weekly	Shöber test; occipital axis. Cervical arrow. Lumbar and dorsal; flexion and extension of spine; Sorensen-Biering test; lateral inclination measurement; measurement of the height of the ASIS and PSIS. Popliteal angle and psoas extensibility.	Sorensen-Biering test: significant improvements in G1 compared to baseline values. Occipital axis: significant difference for G1 compared to baseline values. Measurement of the height of the ASIS and PSIS. Popliteal angle and psoas extensibility: significant improvements in both groups compared to baseline values. In the resto of the variables there were no significant differences.
Navarro-Brazález et al. (2020) [30]	PFD	96	G1 (n = 32): PFMXG2 (n = 32): AHT G3 (n = 32): APFX	PST PF	G1: 48 ± 12 G2: 48 ± 8 G3: 46 ± 8	8 weeks 2 sessions: 45′/week	PFID-20. PDIQ-7. PFM measurement with manometer and dynamometer	G1, G2 and G3 significantly improved the strength of the PFM compared to baseline values. There were no statistically significant differences between groups.
Collado Serra (2014) [23]	EUI and RP	179	G1 (n = 87): APFX G2 (n = 92): control	-	-	G1: 3 weeks before operation/3 months after. Daily PFMT and weekly AHT. G2: 3 months after operation, daily kegels.	24h-PW. ICIQ-QI SF. continence.	Both significantly reduce the duration and severity of SUI after RP compared to baseline values. G1 significantly reduces the duration and severity of SUI after RP compared to G2.
Resende et al. (2010) [24]	PFM and activation TrA	58	G1 (n = 21): PFMX G2 (n = 21): AHT G3 (n = 16): Control	PST	G1: 56.7 ± 10.7 G2: 51.9 ± 7.4 G3: 58.7 ± 0.4)	3 months daily home exercises	MOGS. PERFECT. MVC by SEMG	G1 and G2 there were no significant differences comparing maximum force and contraction with respect to baseline values. IG1 and G2 resistance improved compared to baseline values. G1 and G2 improved significantly in all variables compared to G3.
Au et al. (2019) [25]	PF	37	G1 (n = 19): PFMX control G2 (n = 18): APFX	PST	G1: 61.3 ± 7.3 G2: 63.0 ± 8.5	26 weeks daily.	HRQOL: FACT-P y PORPUS. IPSS y IIEF-5. VD	Week 26. G1 and G2 improved significantly in continence compared to baseline values. There were no significant differences between groups in functional assessment and quality of life. There were no significant differences in erectile function compared to baseline values or between groups.
Moreno-Muñoz et al. (2021) [26]	Postural control and Deep trunk muscles	117	G1 (n = 62): AHT G2 (n = 55): Control	Independent consultant	G1: 44.54 ± 10.4 G2: 46.89 ± 6.59	8 weeks 2 sessions/week	Stabilometric platform based on the Romberg test. Ultrasound image for TrA activation	G1 improved in all variables related to postural control and TrA activation compared to baseline values and compared to G2. G2 did not obtain significant differences compared to baseline values.
Molina-Torres et al. (2022) [27]	UI + PF activation	117	G1 (n = 62): AHT G2 (n = 55): control	Independent consultant	G1: 44.54 ± 10.4 G2: 46.89 ± 6.59	8 weeks G1: 2 sessions/weekG2: NL	MOGS. PFDI-20. PFIQ-7. ICIQ	In week 26, G1 + G2 improved significantly in continence compared to baseline values. There were no significant differences between groups when measuring UI functional assessment and quality of life. There were no significant differences in erectile function compared to baseline values or between groups.
Vicente-Campos et al. (2021) [28]	Diaphragm in NSLBP	40	G1 (n = 20): AHT G2 (n = 20): control	PST	G1: 23.25 ± 4.52 G2: 23.90 ± 7.36	8 weeks G1: 2 HAG training for week. G2: NL G2 (control): NL	Diaphragm thickness. PImax. NRS. Pain threshold. IPAQ. RMDQ. CSI. TSK-11	G1 improved significantly compared to G2 in diaphragm thickness, forced inspiration and decreased scores or central sensitization, disability and pain. There were no significant differences between groups in the rest of the variables. Pre-test and post-test measures were not specified.
Mitova et al. (2022) [29]	PFD	47	G1: Kegel G2: AHT G3: AHT + Kegel	-	G1: 38.53 ± 2.92 G2:39.13 ± 3.09 G3: 39.44 ± 3.12	12 weeks G1: Kegel twice daily. G2: AHT 5 times a week G3: combination of both	UDI-6. IIQ-7 and Kiel test for abdominal, back and hip extensor muscles.	All 3 groups obtained improvements in abdominal strength and resistance, gluteal and back muscles with respect to baseline values. Significant improvements in G2 and G3 in abdominal strength and endurance, gluteal and back with respect to g1. There were no significant differences between G2 and G3. Regarding quality of life all groups improved compared to baseline values, but there were no significant differences between groups. 3 groups obtained improvements in continence compared to baseline values, but only G3 obtained significant improvements both with respect to baseline values as with respect to G1 and G2.

Note: G1: group 1; G2: group 2; G3: group 3; IQR: interquartile range; UI: urinary incontinence; EUI: effort urinary incontinence; PST: physiotherapist; PF: pelvic floor; AHT: abdominal hypopressive technique; PFM: pelvic floor muscles; TrA: transversus abdominis; RP: radical prostatectomy; PFD: pelvic floor dysfunction; NSLBP: nonspecific low back pain; PFMX: pelvic floor muscle exercises; APFX: pelvic floor exercises with hypopressives; PFMT: pelvic floor muscle training; BFB: biofeedback; PFEE: pelvic floor electrostimulation; EMS: electrical muscle stimulation; EMSPF: EMS of pelvic floor; MOGS: Modified Oxford Grading Scale; PERFECT: pelvic floor resistance test; MVC: maximum voluntary contraction; SEMG: surface electromyography; ODI: Oswestry Disability Index; NRS: numerical rating scale; SF-12: 12-item Short Form Health Survey; HRQOL: health-related quality of life; FACT-P: Functional Assessment of Cancer Therapy—Prostate; PORPUS: Patient-Oriented Prostate Utility Scale; IPSS: International Prostate Symptom Score; IIEF-5: 5-item International Index of Erectile Function; VD: voiding diary; ICIQ: International Consultation on Incontinence Questionnaire; ICIQ-SF: ICIQ—Short Form; ICIQ-QI SF: validated Spanish version of the ICIQ; 24h-PW: 24-h Pad Weight test; PFDI-20: Pelvic Floor Distress Inventory—20 items; PFIQ-7: Pelvic Floor Impact Questionnaire—7 items; UDI-6: Urogenital Distress Inventory—6 items; IIQ-7: Incontinence Impact Questionnaire—7 items; RMDQ: Roland-Morris Disability Questionnaire; IPAQ: International Physical Activity Questionnaire; CSI: Central Sensitization Inventory; TSK-11: Tampa Scale of Kinesiophobia—11 items; PImax: maximal inspiratory pressure; FEM: forced expiratory flow; ASIS: anterior superior iliac spine; PSIS: posterior superior iliac spine; LBP: low back pain; NSLBP: nonspecific episodic low back pain; NL: normal life; BPD: bipedestation; STG: sitting; DGD: diaphragmatic global decompression; SD: supine decubitus; DS: dorsal supine; HAG: hypopressive abdominal gymnastics.

**Table 3:** Intervention characteristics.

Authors	Duration	Groups	Learning		Exercise	
Jose-Vaz et al. (2020) [19]	24 sess/12 weeks—50′	AHT	(a) Neutral elongation of pelvis and spine (b) Dorsal ankle flexor (c) Knee flexor (d) Activation of the shoulder girdle muscle (e) 3 respiratory cycles with lateral costal breathing and slow deep exhalation (maximal inhalation-exhalation) (f) Maintenance of breathing after expansion of the rib cage (diaphragmatic aspiration)		First Phase (4 week)	3 sets of 8 repetitions. 3′ between sets Exercises: 1 SD with flx legs + 1 STG with hands on hips, chest and head + 1 cat position
					Second Phase (4 week)	3 sets of 10 reps. 3′ between sets. Exercises: SD with alternating leg flx + 1 STG with legs in front. Hands on hips, chest and overhead + 1 BPD against Wall. Hands on hips, chest and overhead.
					Third Phase (4 week)	3 sets of 12 reps. 3′ between sets. Exercises: 1 on knees+ 1 BPD without support+ 1BPD with flexing the trunk and hand on the Knee. All with hands on hips, chest and above head.
		EMS	Series of 8/12 contractions close to the maximum PFM holding each one for 6” with ¾ contractions at the top.		First Phase (4 week)	3 sets of 8 reps, 6” duration and 3 contractions at the top, 1′ between sets. Exercises: 2 SD + 1 STG
					Second Phase (4 week)	3 sets of 10 reps, 6” duration and 3 contractions at the top, 1′ between sets. Exercises: 1 DS +1 SDT+ 1 BPD
					Third Phase (4 week)	3 sets of 12 reps, 6” duration and 4 contractions at the top, 1′ between sets. Exercises: 3 STG + BPD
Resende et al. (2012) [20]	3 month	APFX	1st date	Information on PF and TrA localization and function. SD with knees in flex., PF localization with a mirror and palpation of the perineum. PF contraction is taught.	2 sets of 8–10 reps daily, contraction held for 6–8” SD and BPD voluntary contraction of the PF with diaphragmatic aspiration. Daily exercises at home for 3 months. Calls every 2 weeks and monthly appointments.	
			2nd date	AHT Learning. Slow DFG inspiration followed by total expiration. Glottic closure and abdominal Wall contraction with diaphragmatic aspiration.		
			3rd date	Home exercise training.		
		EMSPF	1st date	Information on PF localization and function. SD with knees in flx, PF localization with a mirror and palpation of the perineum. PF contraction is taught in the expiratory phase.	3 sets of 8–12 voluntary contractions max held for 6” with 12“of rest between contractions. Then 3 fast contractions in a row. Calls every 2 weeks and monthly appointments.	
			2nd date	Increased PF awareness with vaginal cone; avoiding extraction with voluntary contraction. Stretch reflex by vaginal palpation associated with voluntary PF contraction.		
			3rd date	Home exercise training.		
Dierick et al. (2018) [21]	3 sessions 30′-week for 3 weeks	APFX	(1) Slow and Deep inspiration (2) Complete expiration (3) Diaphragmatic aspiration with apnea of 20 s		3 sets of 8–12 repetitions, BPD exercise, quadruped and SD, with active movements. The sequence of postures is t the discretion of the PST	
		BFB EMSPF			Transvaginal electrical stimulation (excitomotor, bidirectional, rectangular, symmetrical current) through vaginal probes, 15′ (450” in right RP and 450” in left RP) BFB lasting 15′ (6” of muscle contraction and 12” of rest). EMS frequency between 20 and 50 Hz Biphasic pulses of 1 ms Intensity at maximum will up to a maximum of 100 mA.	
Bellido-Fdez et al. (2022) [22]	8 sessions 30′ in 5 weeks	Masotherapy			Spine-focused massage therapy protocol designed for the soft tissues of the thoracic, lumbar and cervical systems, the entire fascial system and vertebral joints.	
		AHT			6 AHT static abdominals. Each exercise is repeated 3 times in addition to a previous Learning phase and a rest period.	
		AHT + Masotherapy			4 massage therapy sessions. 4AHT sessions in alternating sessions.	
Caufriez et al. (2006) [31]	1 a week for 1hour for 10 weeks	AHT	Self-elongation is requested. The patient must bring the elbows outwards in the direction of the longitudinal axis of the arm. While the hands remain at the level of the iliac crests. The patient is instructed to take a lower costal inhalation in such a way that the lower ribs are raised. Then, a full exhalation is requested. Maintaining self-elongation, and finally, perform an inspiratory movement with the glottis closed, emphasizing the abduction of the shoulder blades. Self-elongation and elevation of the lower ribs.		Each posture is held for 15–30” and repeated 3 times, with 20” rest between exercises. Exercises: BPT with hands pointing forward, BPT trunk inclination, semi-sitting, seated-tailor, knees with hands lateral to the iliac crests, quadruped. SD hands lateral to the iliac crests seated-genupectoral.	
		Control			Same positions as AHT but without muscle activation to reduce abdominal pressure.	
Navarro-Brazález et al. (2020) [30]	2 sessions of 45’/week—8weeks	EMSPF	PFMX based on Perfect scheme. 10 fast max contractions of 1” each. Hold contraction for 10”. Repeat sequence 10 times. Manual resistance to improve strength and palpation-based feedback.		If a score of <3 was achieved, intravaginal EEM was used for 15’ to improve awareness and PF contraction. SD exercises with manometric probe.	
					If a score > 3 was achieved or with a large urogenital hiatus, the dynamometry probe was used. They progressed to manometry once they were able to generate pressure while performing the exercises. If the women progressed sufficiently, the last 2 BFB sessions were performed in a more functional standing position.	
					The biofeedback system offered different displays to support concentric, isometric and eccentric exercises; specific exercises and timing were adjusted based on the women’s ability and progressed when appropriate.	
		AHT	Exhale to your expiratory reserve volumen. Then hold your breath (apnea) and expand your rib cage to draw the abdominal Wall inward and cranially without inhaling. Maintain the apnea and expansion of the rib cage for approximately 10”.		3 reps with a breath rest between reps. 3 times rest between reps. 5–10 exercises within each session. Exercises: BPT, kneeling, kneeling on all fours, STG and SD. Using a variety of upper and lower extremity positions.	
		APFX	Same Learning as in the other groups-		Half of the session in EMSPF and half in AHT. Exercises: 2 SD postures, 1 four-point kneeling and 2 BPT.	
Collado-Serra (2013) [23]	3 weeks before prostatectomy and 3 months after.	APFX			AHT: with assessed BFB (with Surface electrodes, periodic PF contractions, 1–3–5”, fast contractions, intense and max strength contractions) and TrA activation. Daily PFMX at home.	
		Control			Oral instructions for Kegel exercises.	
Resende et al. (2010) [24]	12 weeks	AHT	1st date	Lifestyle counseling. Information on PF and TrA location and function. Activation of TrA in SD during full expiration. PF contraction is taught.	2 sets of 8–10 reps daily. Contraction held for 5–8”, 40′ each session. SD and BPT voluntary contraction of the PF with diaphragmatic aspiration. Daily exercises at home for 3 months.	
			2nd date	AHT Learning. SD with knees flx., slow DFG inspiration followed by total expiration. Glottic closure and abdominal Wall contraction with DFG aspiration.		
			3rd date	Home exercise training.		
		EMSPF	1st date	Lifestyle counseling. Information on PF SD location and function with flexed knees. PF localization with a mirror and palpation of the perineum. PF contraction is taught in the expiratory phase.	3 sets of 8–12 voluntary contractions of 6” max with 12” rest between contractions. Followed by 3 fast contractions. SD, STG and BPT exercises.	
			2nd date	Increased PF awareness with vaginal cone; avoiding extraction with voluntary contraction. Stretch reflex by vaginal palpation associated with voluntary PF contraction.		
			3rd date	Home exercise training.		
Au et al. (2019) [25]	26 weeks	AHT + FPilates + EMSPF	Protocol published		Increasing progression every 2 weeks. 30 reps/day during weeks 1–2 up to 180 reps during weeks 7–26.	
		EMSPF	Instructions to isolate and contract the PF muscles to the maximum.		Increasing progression every 2 weeks. 30 reps per day during weeks 1–2 up to 180 reps in weeks 7–26.	
Moreno-Muñoz et al. (2021) [26]	2 sessions of 30′/week—8 weeks.	AHT	First 2 sessions	Low-PressureFitness Protocol Learning respiratory and postural techniques: self-elongation + decoaptation of the glenohumeral + dorsal ankle flexor + overflow of the gravity axis + DGD+ expiratory apnea + costal opening	3 reps with each posture, changing during the expiratory phase. Expiratory apnea with each posture and after 3 respiratory cycles.	
			Rest of sessions	Complete progression of postures from BPT to SD.		
Molina-Torres et al.,(2022) [23]	2 weekly sessions, 20′/8 weeks	AHT	Postural breathing patterns. Rib cage proprioception, familiarization with apnea and abdominal vaccum. Postural indications: axial lengthening + neutral pelvis + projection of the center of gravity + ankle dorsiflexion + activation of the shoulder girdle.		Dynamic progression of AHT in BPD, STG, quadruped and SD.	
Vicente-Campos et al.,(2021) [28]	2 sessions/week, 30–40′/8 weeks	AHT	(a) Neutral pelvis, as well as elongation of the spine (b) Dorsiflexion of the ankles (c) Flx of the knees (d) Shoulder girdle muscle activation (e) 3 breathing cycles completed with costolateral breathing, as well as slow, Deep exhalations (maximal inhalation and exhalation) (f) Maintenance of breathing after expansion of the rib cage (“diaphragmatic aspiration”)		6 AHT separated by 2′ of recovery between them. Each exercise was repeat 3 times.	
Mitova et al. (2022) [29]	12 weeks Kegel: twice daily. AHT: 45′, 5 times/week. AHT + Kegel: both protocols	Kegel	Kegel Methd. Specific exercises were performed starting from initial positions.		Not specified.	
		AHT	Deep breathing in 3 phases. In third, called expiratory apnea, a strong contraction of the abdominal muscles is performed.			
		AHT + Kegel	Both methods were applied.			

Note: SD: standing; STG: standing against the wall; BPD: bipodal position; BPT: bipedal position; DS: sitting; SDT: standing with trunk flexion; PF: pelvic floor; PFMX: pelvic floor muscle exercises; EMSPF: electrostimulation and PF exercises; BFB: biofeedback; TrA: transversus abdominis; AHT: abdominal hypopressive technique; APFX: AHT combined with PFMX; DFG: diaphragmatic breathing; DGD: discharge of the gravity axis; RP: radical prostatectomy; UI: urinary incontinence.

## Data Availability

No new data were created or analyzed in this study. Data sharing is not applicable to this article.

## Abbreviations

The following abbreviations are used in this manuscript:

CORE	Lumbo-Pelvi Coxal Complex
PF	Pelvic Floor
AHT	Abdominal Hypopressive Techniques
PRISMA	Preferred Reporting Items for Systematic Reviews and Meta-analyses
RoB	Risk of Bias
PFMX	Pelvic Floor Muscle Exercise
UI	Urinary Incontinence
TrA	Abdominis Transversus

## Appendix A

**Table A1:** Search equations used in the databases.

Database	Search Equations
PUBMED	((“Pelvic Floor” [Mesh]) OR (“Pelvic Floor Disorders” [Mesh])) AND ((“hypopressive exercise”) OR (“hypopressive abdominals”) OR (“hypopressive method”)) ((“Abdominal Muscles” [Mesh]) OR (“Abdominal CORE” [Mesh])) AND ((“hypopressive exercise”) OR (“hypopressive abdominals”) OR (“hypopressive method”)) ((“Low Back Pain” [Mesh]) OR (“Back Pain” [Mesh]) AND ((“hypopressive exercise”) OR (“hypopressive abdominals”) OR (“hypopressive method”)) (“Diaphragm” [Mesh]) AND ((“hypopressive exercise”) OR (“hypopressive abdominals”) OR (“hypopressive method”))
WOS	(((((TS = (hypopressive method)) OR TS = (hypopressive exercise)) OR TS = (hypopressive abdominals))) AND (((TS = (pelvic floor)) OR TS = (pelvic floor disorders)))) (((((TS = (abdominal CORE)) OR TS = (abdominal muscles))) AND (TS=(hypopressive exercise)) OR TS = (hypopressive abdominals)) OR TS = (hypopressive method)) ((((TS = (low back)) OR TS = (low back pain)) AND (((TS=(hypopressive exercise)) OR TS = (hypopressive method)) AND TS = (hypopressive abdominals)))) (((TS = (diaphragm)) AND (((TS = (hypopressive exercise)) OR TS = (hypopressive method)) OR TS = (hypopressive abdominals))))
PEDro	hypopressive* “pelvic floor*” “pelvic floor disorders*” hypopressive* “low back pain*” “low back*” hypopressive* “abdominal CORE*” “abdominal wall*” hypopressive* diaphragm*
CLINICALTRIALS	hypopressive exercise, hypopressive abdominals, hypopressive method|Pelvic Floor Disorders hypopressive exercise, hypopressive abdominals, hypopressive method|abdominal CORE hypopressive exercise, hypopressive abdominals, hypopressive method|Low Back Pain hypopressive exercise, hypopressive abdominals, hypopressive method|Diaphragm
Cochrane	“HYPOPRESSIVE” AND “PELVIC FLOOR” “HYPOPRESSIVE” AND (“ABDOMINAL MUSCLES” MESH) “HYPOPRESSIVE” AND (“LOW BACK PAIN” MESH) “HYPOPRESSIVE” AND “DIAPHRAGM”
Scopus	(TITLE-ABS-KEY ((“Pelvic Floor” OR “Pelvic Floor Disorders“) AND (“hypopressive abdominals” OR “hypopressive method”)) TITLE-ABS-KEY (((“Abdominal muscles” OR “Abdominal core”) AND (“hypopressive abdominals” OR “hypopressive AND method”))) TITLE-ABS-KEY (((“Low Back Pain” OR “Low Back”) AND (“hypopressive abdominals” OR “hypopressive AND method”))) TITLE-ABS-KEY TITLE-ABS-KEY (((“Diaphragm”) AND (“hypopressive abdominals” OR “hypopressive AND method”)))
